# Prevalence and genotype distribution of human papillomavirus infection among women in northeastern Guangdong Province of China

**DOI:** 10.1186/s12879-018-3105-x

**Published:** 2018-05-03

**Authors:** Pingsen Zhao, Sudong Liu, Zhixiong Zhong, Jingyuan Hou, Lifang Lin, Ruiqiang Weng, Luxian Su, Nanxiang Lei, Tao Hou, Haikun Yang

**Affiliations:** 1 Clinical Core Laboratory, Meizhou People’s Hospital (Huangtang Hospital), Meizhou Hospital Affiliated to Sun Yat-sen University, Meizhou, 514031 People’s Republic of China; 2 Center for Precision Medicine, Meizhou People’s Hospital (Huangtang Hospital), Meizhou Hospital Affiliated to Sun Yat-sen University, Meizhou, 514031 People’s Republic of China; 3 Guangdong Provincial Engineering and Technology Research Center for Molecular Diagnostics of Cardiovascular Diseases, Meizhou, 514031 People’s Republic of China; 4 Meizhou Municipal Engineering and Technology Research Center for Molecular Diagnostics of Cardiovascular Diseases, Meizhou, 514031 People’s Republic of China; 5 Meizhou Municipal Engineering and Technology Research Center for Molecular Diagnostics of Major Genetic Disorders, Meizhou, 514031 People’s Republic of China; 6 Department of Gynaecology, Meizhou People’s Hospital (Huangtang Hospital), Meizhou Hospital Affiliated to Sun Yat-sen University, Meizhou, 514031 People’s Republic of China

**Keywords:** Human papilloma virus (HPV), Cervical cancer, Genotypes, Infection, Southern China

## Abstract

**Background:**

Human papillomavirus (HPV) DNA testing is an important method in cervical cancer screening. However, the studies on prevalence and genotype distribution of HPV among women in northeastern Guangdong Province of China are very limited.

**Methods:**

A total of 28,730 women attending the Department of Gynecology of Meizhou People’s Hospital (Huangtang Hospital), Meizhou Hospital Affiliated to Sun Yat-sen University between January 1st, 2013 and June 1st, 2015 were enrolled in this study. HPV type-specific distribution was tested using flow-through hybridization and gene chip.

**Results:**

The overall prevalence of HPV infection was 19.81%, among which 79.09% were infected with high-risk HPV subtypes in the subjects. The 5 most predominant genotypes were HPV16, 52, 58, 18 and 81. Most HPV infections were observed in women aged 41–50 and women aged 30–59 accounted for a proportion of over 80%.

**Conclusions:**

Our findings suggested a high burden of HPV infection among women in northeastern Guangdong Province of China. We identified the top 5 HPV genotypes as well as the age-specific distribution of HPV infections in this area.

## Background

Human papilloma virus (HPV) is a kind of double-stranded DNA virus without enveloped icosahedral capsids. This virus infects epithelium of cutaneous or mucosal tissues [1]. HPV infection is known as the major causes of cervical cancers, some vaginal, vulvar, and penile cancers. Furthermore, recent evidence has demonstrated that HPV infection at least responsible for 25% of head and neck cancers (HNCs), among which are up to 60% of the oropharyngeal carcinoma (OPC) [2].

As the third most common type of cancers in women, cervical cancer accounts for 9% of the total new diagnosed cancer cases and 8% of the total cancer deaths among females respectively. Notedly, more than 85% of these new cases and deaths occurred in developing countries [3]. Incidence of cervical cancer of females in China is estimated to be about 8.7–11.3/100,000 and the mortality of women with cervical cancer is up to 45.0% [4–6]. It has been established that most cervical cancers are caused by HPV infections [7]. HPV infections are so common that approximately 75% to 80% of sexually active individuals would be infected in their lifetime [8]. The highest prevalence was observed in women under age 25 [9]. The HPV infection rate and HPV genotypes are varied among different countries [10], and even varied in different regions of one countries [11]. It is reported that the global prevalence of HPV infection in women without cervical abnormalities is 11% to 12%, while in Asia as whole and China, this data goes to 8% and 11.4%–20.3%, respectively [10, 12]. This highlights that China is a country with a high burden of HPV infections. As the most important province in southern China, Guangdong has a high HPV burden as its infection rate of high-risk HPV reaches to about 21.07% [4].

Liquid-based cytology is still the most common screening test for HPV infection. Concerns about cytology test lie in its subjective and significant inter-laboratory variation, as well insufficient sensitivity and high false-negative [7, 13]. Currently more and more molecular testing methods have been applied to HPV detection in cervical specimen, with the advantage of higher sensitivity and capable of HPV genotyping [14]. Genotyping has an important role in triaging patients for colposcopy in women who are high-risk HPV positive but have normal cytology [7].

Until now more than 200 HPV genotypes have been identified with more than 2% different homology between each two types [7]. HPVs are commonly divided into high-risk or low-risk based on their capacity to drive the development of cancer. There are at less 12 HPV subtypes, i.e. HPV16, HPV18, HPV31, HPV33, HPV35, HPV39, HPV45, HPV51, HPV52, HPV56, HPV58, and HPV59, that are evidently considered as high-risk and one less evident high-risk is HPV68 [7]. These 13 HPV subtypes have been proved to cause more than 96% cervical cancers [15]. Additionally, another 12 HPV subtypes that have been associated with rare cases of cervical cancer are also defined as high-risk [16]. Low-risk genotypes infection, such as HPV6 and HPV11, cause benign or low-grade changes in cervical cells of genital warts [17]. The process from HPV infections to development of cervical cancer usually takes a long period and only the continued infection of high-risk HPV might result in cervical cancer as well as high-risk HPV raise different risk to cervical cancer [18]. This highlights the importance of HPV genotyping in early detection, treatment as well as prognosis of cervical cancer.

It was shown that a HPV vaccine provides potent protections against HPV infection [19]. To date, there are four prophylactic vaccines targeting various high-risk HPV types, including monovalent vaccine (HPV16), the bivalent vaccine (HPV 16/18), the quadrivalent vaccine (HPV16/18/6/11) and the nonavalent vaccine (HPV 16/18/6/11/31/33/45/52/58) [20]. Therefore, detection and genotyping of HPV is of high importance since it provides valuable information for proper HPV vaccine development.

In the present study, we detected and genotyped HPV infection among women attending Department of Gynecology of Meizhou People’s Hospital (Huangtang Hospital), Meizhou Hospital Affiliated to Sun Yat-sen University during 2013–2015. Our study aimed to assess the prevalence of HPV infection and identify the predominant HPV genotypes in women in northeastern Guangdong Province of China.

## Methods

### Study subjects

Twenty-eight thousand seven hundred thirty women attending Department of Gynecology of Meizhou People’s Hospital (Huangtang Hospital),Meizhou Hospital Affiliated to Sun Yat-sen University between January 1st, 2013 and June 1st, 2015 were enrolled in our study. All of the participants enrolled in this study came from the eight counties of Meizhou city, which is located in the northeastern Guangdong Province of China. Patients were enrolled according to the including criteria: (i) aged 15–89; (ii) didn’t have HPV screen before; (iii) provided enough cervical specimen for HPV DNA testing. This study was approved by the Human Ethics Committees of Meizhou People’s Hospital (Huangtang Hospital), Meizhou Hospital Affiliated to Sun Yat-sen University, Guangdong Province, China. All patients provided written informed consent to participate. For the sake of privacy, data were de-identified before analysis.

### Specimen collection and storage

Cervical specimens were collected by a gynecological practitioner using plastic cervical brushes. All cervical brushes and store bottles with specimen transport medium were from the manufacturer (Hybrobio Biotechnology Corp., Chaozhou, Guangdong, China). In brief, specimen was obtained by inserting the cytobrush into the endocervical canal and rotating four times in a clockwise direction to collect the cervical epithelial cells which adhered to the flat sides of the bristles. The tip of brush was then placed into a vial containing transport medium and stored at 4 °C.

### DNA extraction

DNA from exfoliated cervical cells collected in transport medium was extracted using DNA extraction kit (Hybrobio Biotechnology Corp., Chaozhou, Guangdong, China). Briefly, specimen was added with proteinase K and BL buffer for digestion, followed by ethanol precipitation and column collection. Nanodrop 2000 (ThermoFisher Scientific, CA, USA) was used to determine the concentration and purification of DNA productions.

### Detection and genotyping of HPV

HPV DNA amplification and genotyping were performed using HPV genotyping Kit (Hybrobio Biotechnology Corp., Chaozhou, Guangdong, China) according to the manufacturer’s instructions. Amplification of HPV-DNA was done with the L1 consensus HPV PGMY09/PGMY11 primer set. In brief, the amplification system was consisting of 1 μl HPV-DNA and 24 μl reaction system (Hybribio Biotechnology PCR Kit, Chaozhou, Guangdong, China). HPV was amplified using Roche LightCycler 480 instrument (Roche, USA) following a specific program provided by manufacturer. HPV genotyping was done by flow-through hybridization and gene-chip which contained type-specific oligonucleotides (Hybribio Biotechnology Corp., Chaozhou, Guangdong, China). The gene-chip includes 21 type-specific oligonucleotides designed to detect 15 high-risk(HR) HPV genotypes (HR: 16, 18, 31, 33, 35, 39, 45, 51, 52, 53, 56, 58, 59, 66 and 68) together with 6 low-risk(LR) HPV genotypes (LR: 6, 11, 42, 43, 44 and 81) The final results were determined by colorimetric change on the chip under direct visualization. Quality controls were performed throughout the experiments, including DNA extracting, amplification and hybridization by applying positive and negative controls for PCR reaction.

### Data analysis

Proportion of women in different age groups in each year was presented. Single, double and multiple HPV infection was defined as infected by one, two or more than two subtypes of HPV infections. Type-specific HPV positivity and corresponding 95% confidence interval (CI) was calculated. Descriptive and inferential statistical analysis of the data was done using Statistical Package for the Social Sciences version 22 (SPSS Inc., Illinios, USA). Data were compared by Pearson χ^2^ or Fisher exact tests as appropriate. *P* < 0.05 was considered to be statistically significant.

## Results

### Participants and prevalence of HPV infection

**Fig. 1:**
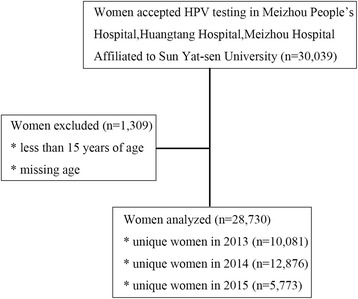
Flow chart of study subject inclusion and exclusion

As shown in Fig. 1, between January 1st, 2013 and June 1st, 2015, there were 30,039 women attending Department of Gynecology of Meizhou People’s Hospital (Huangtang Hospital), Meizhou Hospital Affiliated to Sun Yat-sen University that accepted HPV DNA testing. One thousand three hundred nine women were excluded from our study because they were either under the age of 15 or without age information. Finally, this study included 28,730 participants for analysis.

A total of 5690 (19.81%) women were infected with HPV in this period. 4500 (15.66%) women had HR infection and 678 (2.36%) women have LR infection and 512 (1.78%) women had both HR and LR HPV infection. Meanwhile, double and multiple HPV infections were observed with infection rates of 3.84% and 0.66%, respectively (Table 1).

**Table 1:** Prevalence of HPV infection in study participants in northeastern Guangdong Province of China

	Single	Double	Multiple	Total
Negative	–	–	–	23,040 (80.19%)
Positive	4396 (15.30%)	1104 (3.84%)	190 (0.66%)	5690 (19.81%)
HR only	3747 (13.04%)	621 (2.16%)	132 (0.46%)	4500 (15.66%)
LR only	649 (2.26%)	27 (0.09%)	2 (0.00%)	678 (2.36%)
HR&LR	–	456 (1.59%)	56 (0.20%)	512 (1.78%)

HR:high-risk; LR:low-risk

### HPV genotype distribution

Twenty-one different HPV subtypes were identified in this study. HPV16,52 and 58 were the three most predominant subtypes, with proportions of 27.81%, 14.60% and 10.58%, respectively. Other genotypes that ranked top ten were HPV18, 81, 53, 33, 31, 51 and 39, with corresponding proportions of 6.01%, 5.45%, 5.10%, 4.94%, 3.80%, 3.69% and 3.28% (Table 2). To be noted, HPV81 is the only LR subtype that has a relative high infection rate in women. Double or multiple HPV infections were common, especially among the HR subtypes like HPV16, 18 and 33.

**Table 2:** Genotypes of the HPV infection in study participants in northeastern Guangdong Province of China

HPV Type	Single	Double	Multiple	Total
6	136 (2.63%)	11 (0.21%)	1 (0.02%)	148 (2.86%)
11	136 (2.63%)	4 (0.08%)	1 (0.02%)	141 (2.72%)
16	1121 (21.65%)	245 (4.73%)	74 (1.43%)	1440 (27.81%)
18	237 (4.58%)	60 (1.16%)	14 (0.27%)	311 (6.01%)
31	143 (2.76%)	48 (0.93%)	6 (0.12%)	197 (3.80%)
33	201 (3.88%)	42 (0.81%)	13 (0.25%)	256 (4.94%)
35	23 (0.44%)	7 (0.14%)	2 (0.04%)	32 (0.62%)
39	136 (2.63%)	28 (0.54%)	6 (0.12%)	170 (3.28%)
42	23 (0.44%)	3 (0.06%)	0	26 (0.5%)
43	7 (0.14%)	2 (0.04%)	0	9 (0.17%)
44	66 (1.27%)	6 (0.12%)	1 (0.02%)	73 (1.41%)
45	34 (0.66%)	5 (0.10%)	2 (0.04%)	41 (0.79%)
51	145 (2.80%)	40 (0.77%)	6 (0.12%)	191 (3.69%)
52	662 (12.78%)	90 (1.74%)	4 (0.08%)	756 (14.60%)
53	237 (4.58%)	26 (0.50%)	1 (0.02%)	264 (5.10%)
56	47 (0.91%)	8 (0.15%)	1 (0.02%)	56 (1.08%)
58	534 (10.31%)	12 (0.23%)	2 (0.04%)	548 (10.58%)
59	37 (0.71%)	1 (0.02%)	0	38 (0.73%)
66	89 (1.72%)	5 (0.10%)	0	94 (1.82%)
68	141 (2.72%)	4 (0.08%)	1 (0.02%)	146 (2.82%)
cp8304	281 (5.43%)	1 (0.02%)	0	282 (5.45%)

It was shown in Table 3 the HPV type-specific positivity in each year from 2013 to 2015. It is obviously that there is an increasing trend of HPV positivity, as well as HR and LR HPV infections during this period.

**Table 3:** Positivity of HPV infection in study participants in northeastern Guangdong Province of China

HPV type	2013(*N* = 10,081)		2014(*N* = 12,876)		2015(*N* = 5773)		*P*
	n	%(95%CI)	n	%(95%CI)	n	%(95%CI)	
6	38	0.38 (0.26–0.50)	57	0.44 (0.33–0.56)	54	0.94 (0.69–1.18)	< 0.001
11	45	0.45 (0.32–0.58)	65	0.50 (0.38–0.63)	38	0.66 (0.45–0.87)	0.085
16	401	3.98 (3.60–4.36)	626	4.86 (4.49–5.23)	404	7.00 (6.34–7.66)	< 0.001
18	91	0.90 (0.72–1.09)	136	1.06 (0.88–1.23)	113	1.96 (1.60–2.31)	< 0.001
31	54	0.54 (0.39–0.68)	93	0.72 (0.58–0.87)	80	1.39 (1.08–1.69)	< 0.001
33	94	0.93 (0.74–1.12)	132	1.03 (0.85–1.20)	89	1.54 (1.22–1.86)	0.001
35	6	0.06 (0.01–0.11)	13	0.10 (0.05–0.16)	20	0.35 (0.16–0.50)	< 0.001
39	41	0.41 (0.28–0.53)	115	0.89 (0.73–1.06)	69	1.20 (0.91–1.48)	< 0.001
42	4	0.04 (0.00–0.08)	7	0.05 (0.01–0.09)	18	0.31 (0.17–0.46)	< 0.001
43	0	0.00	3	0.02 (0.00–0.05)	7	0.12 (0.03–0.21)	< 0.001
44	15	0.15 (0.07–0.22)	32	0.25 (0.16–0.33)	29	0.50 (0.32–0.68)	< 0.001
45	22	0.22 (0.13–0.31)	25	0.19 (0.12–0.27)	10	0.17 (0.07–0.28)	0.529
51	10	0.10 (0.04–0.16)	104	0.81 (0.65–0.96)	131	2.27 (1.89–2.65)	< 0.001
52	254	2.52 (2.21–2.83)	388	3.01 (2.72–3.31)	248	4.30 (3.77–4.82)	< 0.001
53	99	0.98 (0.79–1.17)	165	1.28 (1.09–1.48)	103	1.78 (1.44–2.13)	< 0.001
56	14	0.14 (0.07–0.21)	42	0.33 (0.23–0.42)	27	0.47 (0.29–0.64)	< 0.001
58	214	2.12 (1.84–2.40)	359	2.79 (2.50–3.07)	200	3.46 (2.99–3.94)	< 0.001
59	18	0.18 (0.10–0.26)	29	0.23 (0.14–0.31)	18	0.31 (0.17–0.46)	0.096
66	31	0.31 (0.20–0.42)	54	0.42 (0.31–0.53)	59	1.02 (0.76–1.28)	< 0.001
68	89	0.88 (0.70–1.07)	87	0.68 (0.53–0.82)	39	0.68 (0.46–0.89)	0.095
cp8304	76	0.75 (0.59–0.92)	117	0.91 (0.74–1.07)	103	1.78 (1.44–2.12)	< 0.001
6/11	83	0.82 (0.65–1.00)	120	0.93 (0.77–1.10)	89	1.54 (1.22–1.86)	< 0.001
16/18	484	4.80 (4.38–5.22)	755	5.86 (5.46–6.27)	510	8.83 (8.10–9.57)	< 0.001
4vHPV	567	5.625.17–6.07)	875	6.80 (6.36–7.23)	599	10.38 (9.59–11.16)	< 0.001
9vHPV	1123	11.14 (10.53–11.75)	1706	13.25 (12.66–13.84)	1080	18.71 (17.70–19.71)	< 0.001
HR HPV	1106	10.97 (10.36–11.58)	1651	12.82 (12.24–13.40)	964	16.70 (15.74–17.66)	< 0.001
LR HPV	169	1.68 (1.43–1.93)	267	2.07 (1.83–2.32)	216	3.74 (3.25–4.23)	< 0.001
ANY HPV	1433	14.21 (13.54–14.90)	2248	17.46 (16.80–18.11)	1499	25.97 (24.83–27.10)	< 0.001

4vHPV: 4-valent HPV types including HPV 6, 11, 16, and 18; 9vHPV: 9-valent HPV types including HPV 6, 11, 16, 18, 31, 33, 45, 52, and 58; HR HPV: high risk HPV types including HPV 16, 18, 31, 33, 35, 39, 45, 51, 52, 53, 56, 58, 59, 66, 68, and cp8304; LR HPV: low risk HPV types including HPV 6, 11, 42, 43, and 44. Any HPV: any of the 21 HPV types mentioned above

### HPV positivity and age

Here we identified the age distribution of HPV infection in a population in northeastern Guangdong Province of China. As shown in Table 4, most HPV infections occurred in women with the age groups of 40–49, 30–39 and 50–59, which in total account for over 80% of the positivity. Besides, the young women aged 20–29 also emerged as a main population that threatened by HPV prevalence of 12.1%.

**Table 4:** Age distribution in study participants in northeastern Guangdong Province of China

Age (year)	2013(N = 10,081)		2014(N = 12,876)		2015(N = 5773)		HPV positivity(*N* = 5180)	
	n	%	n	%	n	%	n	%(95%CI)
15–19	21	0.21	37	0.29	30	0.51	24	0.46 (0.28–0.64)
20–29	1015	10.07	1226	9.52	587	10.17	627	12.10 (11.21–12.99)
30–39	2801	27.79	3407	26.46	1500	25.98	1187	22.92 (21.78–24.06)
40–49	4456	44.20	5758	44.72	2578	44.65	2085	40.25 (38.91–41.59)
50–59	1366	13.55	1867	14.50	817	14.15	906	17.49 (16.46–18.52)
60–69	302	3.00	443	3.44	194	3.35	260	5.02 (4.43–5.61)
70–79	101	1.00	120	0.93	50	0.86	78	1.51 (1.18–1.84)
80–89	18	0.18	21	0.16	18	0.32	13	0.25 (0.11–0.39)

**Fig. 2:**
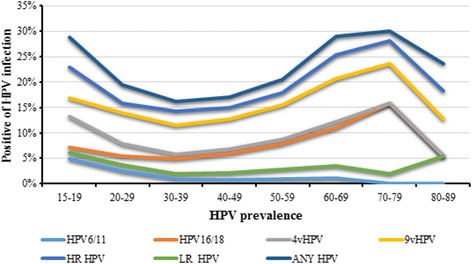
Positivity of type-specific cervical HPV infection in study participants in northeastern Guangdong Province of China. By age group 4vHPV: 4-valent HPV types including HPV 6, 11, 16, and 18; 9vHPV: 9-valent HPV types including HPV 6, 11, 16, 18, 31, 33, 45, 52, and 58; HR HPV: high risk HPV types including HPV 16, 18, 31, 33, 35, 39, 45, 51, 52, 53, 56, 58, 59, 66 and 68; LR HPV: low risk HPV types including HPV 6,11, 42, 43, 44 and 81. Any HPV: any of the 21 HPV types mentioned above

On the other hand, the positivity of HPV types varied in different age groups. As shown in Fig. 2, U-shaped curves were seemed in HPV prevalence that correlated with age group. The positive of combined HPV infection peaked among women aged 15–19 years, then decreased with age, stabilized among women aged 25–49, and then surged again among women aged 50 years and older.

## Discussion

The present study investigated the prevalence of HPV infection and genotypes of HPV in women attending Department of Gynecology of Meizhou People’s Hospital (Huangtang Hospital), Meizhou Hospital Affiliated to Sun Yat-sen University from January 1st, 2013 to June 1st, 2015. To our knowledge, this is the first large-scale study on prevalence and genotype distribution of HPV participants in northeastern Guangdong Province of China.

Type-specific persistent infection, such as HPV16 and 18, has been recognized as a major risk factor for cervical cancer [21]. Cervical cancer is the third common cancer in women worldwide and cause most deaths in developing countries [3]. It was reported previously that cervical cancer was the second leading cause of cancer mortalities among 15- to 44-year-old females in China [22]. In the present study, our data shows that more than 80% HPV infections occurred in women aged 20- to 59-years. This highlights the emergent necessity for these women to be protected from HPV infections. Recent years a predominant reduction of cervical cancer has been achieved in developed countries with the application of effective cervical screening programs [23, 24]. Molecular testing of hrHPV in cervical specimen have served as a complement approach for the early diagnosis of cervical cancer with the advantages of higher sensitivity and specificity [23]. It has been reported that several European countries would carry on hrHPV testing as the primary screen for cervical cancer in view of clinical trials [25].

In addition to HPV screening, HPV vaccination has been shown to be an effective strategy against HPV infection and has been recently implemented in most western countries [20]. To date, there are four HPV vaccines available to protect against HPV, including monovalent vaccine (HPV16), bivalent vaccine (HPV16/18), quadrivalent vaccine (HPV16/18/6/11) [26] and nonavalent vaccine (HPV16/18/6/11/31/33/45/52/58) [27]. Although bivalent vaccine (HPV16/18) and quadrivalent vaccine (HPV6/11/16/18) protect against infection by HPV16 and HPV18, they have little effect on other hrHPVs found in at least 25% of cervical cancers [28].

Our study revealed a 19.81% prevalence of cervical HPV infection among women who presented for cervical cancer screening in Meizhou City of southern China. Wang et al. have studied the nationwide HPV prevalence and HPV genotype distribution in China and found that total hrHPV infection rate in Guangdong Province was 21.07% [4]. However, even in Guangdong Province, the prevalence of HPV infection varied greatly in different cities. The available data showed that the prevalence of HPV infection in Guangzhou, Shenzhen and Chaozhou was 10% [29], 18.4% [30] and 12.6% [31], respectively. Meanwhile, a latest study reported that the HPV prevalence in Hakka women in Heyuan City which is not far away from Meizhou is 12.27% [32]. Several risk factors contribute to the prevalence of HPV, like genetic variation, sexual behavior and biological predisposition [33]. A limitation of our study is that the samples were collected from women attending gynecological department of the hospital. Thus, the rate of HPV positivity might be higher than those in general population. Therefore, our findings might overestimate the prevalence of HPV infection in general population in northeastern region of Guangdong Province in China. Meanwhile, of the 21 HPV types we detected, HPV 16, 52 and 58 were observed to be the most predominant high-risk types among women in this area, which totally accounted for over a half of the HPV infections. This is consistent with the studies generated by some Chinese population-specific investigations [4, 34]. These findings suggested that in addition to HPV16 and HPV18, HPV vaccines against HPV52 and HPV58 would be valuable for population in northeastern Guangdong Province of China.

Meta-analysis showed that global HPV prevalence was higher in the younger and older women [7]. Also, previous studies showed that HPV prevalence in cities of China like Kunming, Hongkong and Heyuan presented the similar pattern. In the present study, we observed peaks of prevalence in subjects aged 15–19 or 60–79 years and steady decrease in mid-aged women. The exact mechanism underlying the relation between HPV infection and age remains unclear. A possible reason may be that the women of these ages have a lower level of hormone as well as weaker immune function and therefore less capable to resist HPV infection [32]. Also, younger women that shortly after becoming sexually active are susceptible to HPV infection because they don’t know how to protect themselves [7]. Besides, this study presented the age-specific HPV prevalence in women. The results showed that more than 40% of HPV infections occurred in women aged 40–49. On one hand, this may be attributed to largest number of participants of these ages. On the other, this also suggested that women of these ages suffer severely from HPV infection As the first study to report year- and age-specific HPV positivity in such a large number of participants in Meizhou, southern China, the information presented in this study would be instructive to protect women against HPV virus.

The prevalence of HPV infection and viral genotypes are different in different nations [4]. Common risk factors that correlate the prevalence of HPV infection include genetic variation, sexual activity (earlier age at first sexual activity, two or more sex partners), coinfection with other pathogens like herpes simplex virus, and human immunodeficiency virus (HIV) [33, 35]. Meizhou is located in northeastern Guangdong Province of China, with a total area of 15,836 km^2^ and a population of 5.05 million. More than 95% people lived in Meizhou are Hakka, which is a special Han Chinese population that have a long history and unique culture, such as language, behavior and traditional food [36, 37]. Currently, few data are available on the prevalence and genotype distribution of HPV infection in Hakka population of Meizhou. Hence, the present study would be instructive to assess the clinical benefits of screening and vaccination strategies against HPV for in this area.

## Conclusions

In conclusion, this study presented the heavy burden of HPV infection and the viral genotype distribution in women in northeastern Guangdong Province of China. HPV16, 52 and 58 are the dominant high-risk genotypes and over 80% of HPV infections occurred in women aged 20–49. The findings in this study provide important information for cervical screening and vaccination in population in northeastern Guangdong Province of China.

## Abbreviations

HIV: Human immunodeficiency virus
HNC: Head and neck cancers
HPV: Human papillomavirus
HR: High-risk
LR: Low-risk
OPC: Oropharyngeal carcinoma
